# Filling the Gap after CDK4/6 Inhibitors: Novel Endocrine and Biologic Treatment Options for Metastatic Hormone Receptor Positive Breast Cancer

**DOI:** 10.3390/cancers15072015

**Published:** 2023-03-28

**Authors:** Abhenil Mittal, Consolacion Molto Valiente, Faris Tamimi, Ilana Schlam, Sarah Sammons, Sara M. Tolaney, Paolo Tarantino

**Affiliations:** 1 Division of Medical Oncology and Hematology, Princess Margaret Cancer Center; Toronto, ON M5G 2C1, Canada; 2 Department of Medicine, University of Toronto, Toronto, ON M5G 2C1, Canada; 3 Department of Hematology and Oncology, Tufts Medical Center, Boston, MA 02111, USA; 4 Department of Medical Oncology, Dana-Farber Cancer Institute, Boston, MA 02115, USA; 5 Harvard Medical School, Boston, MA 02115, USA; 6 Department of Oncology and Onco-Hematology, University of Milan, 20122 Milan, Italy

**Keywords:** breast cancer, CDK4/6-inhibitors, SERD, capivasertib, elacestrant, camizestrant

## Abstract

**Simple Summary:**

There are limited treatment options beyond chemotherapy for patients with hormone receptor positive metastatic breast cancer after progression on first line therapy with CDK4/6 inhibitors and endocrine therapy. Recently, encouraging evidence has emerged from multiple drugs in this space with the potential to delay chemotherapy and improve outcomes. The most promising agents include the AKT inhibitor capivasertib, the oral selective estrogen receptor degrader (SERD) elacestrant, and PARP inhibitors for patients harboring pathogenic germline BRCA1/2 mutations. Additionally, a subset of patients may also potentially be candidates for continuation of CDK4/6 inhibitors beyond progression. In this review, we highlight clinical data supporting the use of these agents and critically analyze the available evidence for their use. We also provide an algorithm to guide clinicians in their daily practice for patients with progression following first line CDK4/6 inhibitors and endocrine therapy.

**Abstract:**

The rise of cyclin-dependent kinase (CDK)4/6 inhibitors has rapidly reshaped treatment algorithms for hormone receptor (HR)-positive metastatic breast cancer, with endocrine treatment (ET) plus a CDK4/6-inhibitor currently representing the standard of care in the first line setting. However, treatment selection for those patients experiencing progression while on ET + CDK4/6-inhibitors remains challenging due to the suboptimal activity or significant toxicities of the currently available options. There is also a paucity of data regarding the efficacy of older regimens, such as everolimus + exemestane, post-CDK4/6 inhibition. In this setting of high unmet need, several clinical trials of novel drugs have recently reported encouraging results: the addition of the AKT-inhibitor capivasertib to fulvestrant demonstrated a significant improvement in progression-free survival (PFS); the oral selective estrogen receptor degrader (SERD) elacestrant prolonged PFS compared to traditional ET in a phase 3 trial, particularly among patients with detectable ESR1 mutations; finally, PARP inhibitors are available treatment options for patients with pathogenic BRCA1/2 germline mutations. Overall, a plethora of novel endocrine and biologic treatment options are finally filling the gap between first-line ET and later line chemotherapy. In this review article, we recapitulate the activity of these novel treatment options and their potential role in future treatment algorithms.

## 1. Introduction

Breast cancer is the most commonly diagnosed cancer in women worldwide and represents the second most common cause of cancer related death in women in the United States, with approximately 43,600 deaths reported in 2021 [1,2]. Despite significant advancements in cancer treatments, patients with metastatic breast cancer (MBC) remain incurable, with a median overall survival (OS) of approximately five years with CDK4/6 inhibitor based therapy [2,3]. About 70% of all breast cancers express the estrogen receptor (ER), the progesterone receptor (PR), or both [4]. These tumors are often sensitive to hormonal manipulation with various drugs including selective estrogen receptor modulators (SERMs) like tamoxifen, aromatase inhibitors (AI) like letrozole, anastrozole and exemestane and selective estrogen receptor degraders (SERDs) like fulvestrant.

Since the first report of efficacy of the cyclin dependent kinase 4/6 (CDK4/6) inhibitor palbociclib in hormone receptor (HR)-positive advanced breast cancer (ABC) [5], multiple large randomized phase three trials have established the efficacy of CDK4/6 inhibitors (palbociclib, ribociclib and abemaciclib) in the first-line setting in both pre-menopausal and post-menopausal women in combination with ET [6,7,8,9,10,11]. OS benefit has been demonstrated for ribociclib plus letrozole/fulvestrant for postmenopausal women, with a median OS of greater than five years in patients receiving the CDK4/6 inhibitor [10,11]. The OS data from the second interim analysis of abemaciclb from the MONARCH-3 trial showed numerical improvement in the experimental arm (67.1 months vs. 54.2 months, hazard ratio 0.754, 0.57–0.97, *p* = 0.0301) but did not meet the pre-specified boundary for statistical significance at this timepoint; the final OS analysis is anticipated later in 2023 [12] Ribociclib in combination with endocrine therapy (ET) has also demonstrated an improvement in OS in premenopausal women and is considered the standard of care in first line setting [9]. Although the relative benefit in progression free survival (PFS) is similar in all first line studies of different CDK4/6 inhibitors with ET, recent survival data from PALOMA-2 trial demonstrated no significant OS benefit with palbociclib [13]. Whether this is related to differential efficacy of different CDK4/6 inhibitors or due to differences in patient population or loss of many patients for survival follow-up remains an open question. Given these findings, ribociclib recently received a category 1 recommendation for first line treatment for women with HR-positive MBC in the NCCN guidelines, whereas palbociclib and abemaciclib still have a category 2A recommendation [14] For patients who have endocrine sensitive disease (de-novo metastatic disease or progression > 12 months after completing adjuvant endocrine therapy), either AI or fulvestrant as ET partner is reasonable given similar efficacy in the PARSIFAL study [15]; however, AI is preferred in the clinic due to oral administration and more data for fulvestrant post progression on AI. For patients with endocrine resistant disease, fulvestrant is the preferred ET partner with a CDK4/6 inhibitor [11,16,17,18].

Eventually, most patients develop resistance to CDK4/6 inhibitors and require a change in therapy. PFS on first line CDK4/6 inhibitor ranges from 2–3 years; however, the median OS of about five years suggests limited efficacy of subsequent anti-cancer treatments. For instance, several recent trials have shown that fulvestrant achieves a median PFS of 2–3 months after progression to CDK4/6 inhibitors, warranting the development of better treatment strategies to extend the endocrine treatment window before moving to cytotoxic chemotherapy [19,20,21] Importantly, in the past few years, the mechanisms of resistance to CDK4/6 inhibitors and endocrine therapy are starting to be unraveled, allowing for an expansion in the pipeline of effective agents in this setting [22,23]. Multiple randomized phase 2 and 3 trials have recently reported positive results, leading to significant changes in treatment algorithms for HR-positive MBC.

In this article, we will review the current practice patterns beyond first line therapy in HR-positive ABC, common resistance mechanisms to CDK4/6 inhibitors and ET, describe the rationale and data for continuation of CDK4/6 inhibitors beyond progression and also delve into the novel endocrine and biological treatment options which may bridge the gap between first line CDK4/6 inhibitors + ET and subsequent chemotherapy for which data have been published or presented in the last few years. Finally, we will discuss how these treatments could be incorporated into future treatment algorithms. 

## 2. Current Practice Standards after Progression on 1st Line CDK4/6 Inhibitor and ET

Data on therapies after progression on 1st line CDK4/6 inhibitor from major randomized trials show that single agent ET was the most commonly pursued strategy (50–60%) followed by chemotherapy (30–35%), mTOR inhibition with everolimus and exemestane (10–15%) and continuation of a CDK4/6 inhibitor (<10%) [24]. Real world studies suggest higher use of chemotherapy in the second line setting compared to a different endocrine strategy, possibly reflecting a fear among oncologists of loss of endocrine sensitivity [25,26,27,28]. Short progression free survival (PFS) under three months with ET monotherapy control arms including fulvestrant in recent randomized and single arm trials provides further credibility to this hypothesis and suggests an urgent need for alternative therapeutic approaches in this patient population [19,20,21].

## 3. Mechanisms of Resistance to CDK4/6 Inhibitors and Endocrine Therapy

Multiple mechanisms of resistance to CDK4/6 inhibitors have been described, including increased activity of the CDK4/6 checkpoint kinase, bypassing the checkpoint through activation of CCNE1/CDK2 leading to downstream phosphorylation of retinoblastoma (RB) protein or acquired RB1 loss of function mutations [29,30]. Several retrospective studies and post-hoc analysis of randomized trials have demonstrated that 5–10% of patients develop acquired RB1 mutation as a mechanism of resistance to CDK4/6 inhibitors, whereas these are a less common cause of intrinsic drug resistance (0–5%) [31,32,33,34,35]. Other mechanisms including c-MET mutations [36], aberrant cyclin E1 signaling [37], CDK6 amplification [38], loss of FAT1 [39] and activation of tyrosine kinase receptor signaling including the PI3K/AKT/mTOR pathway [40]. Resistance to ET (primarily AI) is often mediated by mutations in the alpha subunit of the ER (ESR1 driver mutations) in an endocrine dependent manner or due to constitutive activation of the PI3K/AKT/mTOR pathway in an endocrine independent manner [31,41,42,43]. About 30–40% of patients develop an ESR1 mutation while on treatment with a CDK4/6 inhibitor plus AI, reflecting endocrine resistance; these patients may still retain sensitivity to CDK4/6 inhibition, providing rationale for maintaining CDK4/6 inhibitor beyond progression and targeting ESR1, through a switch in endocrine therapy to a SERD with activity against this mutation [31,32,33,34,35,36,37,38,39,40,41,42,43,44]. Moreover, various drugs targeting the upstream pathways [(PI3 kinase (alpelisib), AKT (capivasertib) and mTOR (everolimus)] have shown clinical benefit in randomized controlled trials and will be discussed below.

## 4. Continuation of CDK4/6 Inhibitors beyond Progression

### 4.1. CDK4/6 Inhibitors plus ET

Potential benefit for continuation of a CDK4/6 inhibitor beyond initial progression was initially demonstrated in retrospective studies; however, most of these studies were small single institutional studies with heterogenous patient populations with heavily pre-treated patients, thus not allowing for any firm conclusions [45,46,47,48]. A larger multicentric study from six academic centers in the United States of abemaciclib post progression on palbociclib showed a median PFS of 5.6 months and median OS of 17.2 months [32].

Prospective data on this strategy are derived from the randomized phase II MAINTAIN trial [21], which enrolled 120 patients with HR-positive MBC. In this study, patients that progressed on prior CDK4/6 inhibitor plus ET were randomized to ribociclib plus switch of ET vs. placebo plus switch ET. Notably, most patients received an AI as initial ET, 83% of the patients received palbociclib as initial CDK4/6 inhibitor and more than two thirds had previously received a CDK4/6 inhibitor for >12 months. A small number of patients had received chemotherapy for MBC (around 10%). The study was powered for PFS as the primary end point which was met with an approximately 2.5 months improvement in PFS in the ribociclib plus ET arm compared to the placebo arm (median 5.29 months vs. 2.76 months, hazard ratio 0.57 (0.39–0.95), *p* = 0.006). The objective response rate (ORR) with ribociclib was 20% compared to 11% with ET alone. Data on OS and safety are not yet available [21]. In an exploratory analysis, there was no benefit in patients who had an *ESR1* mutation at study entry with equally poor outcomes in both arms (median PFS 3 months). However, this analysis is limited by small numbers of patients with an ESR1 mutation (*n* = 33) and higher number of patients with CCND1 (24%) and FGFR1 (9%) alterations among the ESR1 mutant cohort, which might have limited the benefit of CDK4/6 inhibitor continuation in this subgroup [21].

The second prospective trial in this space for which results have been recently reported is the PACE phase two randomized study. Patients in this study were randomized to palbociclib plus fulvestrant vs. placebo plus fulvestrant, with the comparison among these two arms being the primary end point. A third arm tested triple therapy with palbociclib, fulvestrant and avelumab. The study enrolled a similar patient population to the MAINTAIN trial, with the majority of patients having received prior palbociclib as the CDK4/6 inhibitor (>90%) with duration of exposure of >12 months (76%) [49]. More than half of patients had visceral disease (60%), and only a minority had received prior chemotherapy for MBC (16%). Contrary to the findings from MAINTAIN, there was no benefit for continuing palbociclib beyond progression in terms of either PFS (median PFS 4.6 months vs. 4.8 months, hazard ratio 1.11 (0.74–1.66) or OS (median OS 24.6 months vs. 27.5 months, hazard ratio 1.02 (95% CI 0.67–1.56)). Again, contrary to MAINTAIN data, there was a trend towards PFS benefit in patients who had an ESR1 mutation with combination but no benefit in those who were ESR1 wild type. However, these analyses were exploratory.

Given the conflicting results of two prospective studies and the phase two nature of these trials, the benefits of continuing CDK4/6 inhibitor beyond progression remain controversial. It is plausible that switch to a different CDK4/6 inhibitor as tested in the MAINTAIN trial may be worthwhile in some patients rather than continuing the same agent, as was done in PACE. There are known biological and pharmacological differences among CDK4/6 inhibitors which might contribute to different mechanisms of resistance and differential efficacy in this setting [50]. Of note, the NATALEE phase 3 trial of adjuvant ribociclib was recently announced to meet its primary endpoint of invasive disease free survival, whereas Palbociclib failed to improve outcomes in two trials conducted in a similar setting (PALLAS and PENELOPE-B). Moreover, none of the maintenance studies tested a switch to abemaciclib, which has the highest single agent activity of all CDK4/6 inhibitors in a heavily pre-treated MBC population [51], and can also be modestly improved by adding tamoxifen to abemaciclib as seen in the nextMONARCH trial [52]. In the single arm Phase II ELAINE II study, abemaciclib and the novel selective estrogen receptor modulator lasofoxifene were trialed post-CDK4/6 inhibitors in 29 patients harboring ESR1 mutations. The median PFS was 13.9 months with an ORR of 33.3% (ASCO 2022) [53].

Ongoing randomized trials are likely to provide further evidence into this critical question. The postMONARCH phase 3 trial will evaluate the role of adding abemaciclib to fulvestrant in patients that have experienced progression to CDK4/6 inhibition. The EMBER 3 phase 3 trial will evaluate the novel SERD imlunestrant, alone or with abemaciclib, compared to investigator choice of endocrine treatment. The PALMIRA study will look at palbociclib rechallenge (similar to PACE) but in a population that has previously documented clinical benefit to palbociclib; this study has completed accrual and results are pending [54]. A similar single arm phase II study ongoing in Italy is currently recruiting [55]. ELAINE 3 will evaluate lasofoxifine/abemaciclib versus fulvestrant/abemaciclib in patients with prior progression on ribociclib or Palbociclib. A phase 1/2 study looking at dual CDK2 and CDK4/6 inhibitor after progression on CDK4/6 inhibitors in multiple tumor types is also ongoing and addresses a key resistance mechanism to these agents [56] (Table 1).

### 4.2. CDK4/6 Inhibitors plus Other Targeted Agents including Immunotherapy

Pre-clinical evidence suggests an interaction between checkpoint inhibitors and CDK4/6 inhibitors which might be mediated through programmed death-ligand 1 (PD-L1) degradation by SPOP or direct stimulation of PD-L1 expressing T cells by CDK 4/6 inhibitors [68,69], thus providing rationale for testing combination strategies using these drugs. One arm of the PACE study mentioned above tested the PD-L1 inhibitor avelumab in combination with palbociclib and fulvestrant in patients previous treated with palbociclib [49]. Superiority of this triplet over fulvestrant alone was a secondary end point of the study. Interestingly, both PFS and OS with triplet regimen were numerically longer in the PACE trial than either fulvestrant or combination of palbociclib plus fulvestrant, although the differences were not statistically significant. No major toxicity signals were identified, different from previous studies that have found concerning toxicities when CDK4/6 inhibitors and immune checkpoint inhibitors have been combined [70,71,72]. Overall, this was a small trial, underpowered to detect this difference and this observation is hypothesis generating and warrants evaluation in future trials. The approach is current being tested with atezolizumab in different combinations with other targeted agents [57] (Table 1). 

Synergistic activity for CDK4/6 inhibitors with PI3K or mTOR inhibitors has also been observed and is currently being tested in clinical trials [73]. One of the first studies testing this concept using a combination of palbociclib with everolimus and exemestane was limited by severe toxicity (including high grade mucositis and neutropenia) and limited efficacy [74]. Similar toxicity concerns were observed in another study looking at ribociclib in combination with everolimus and exemestane [75]. This is consistent with the poor tolerability often observed with everolimus in this setting [76]. Other combinations including those effecting upstream signaling [58] or having dual mTOR/PI3K inhibitor activity [59] are being explored. Clinical trials are also testing newer generation FGFR inhibitors like erdafitinib [60] (Table 1). Data on these targeted approaches are awaited with interest and are likely to influence management of patients who have previously progressed on CDK4/6 inhibitors.

## 5. Fulvestrant and Oral SERDs

The benefit of fulvestrant as second line ET was established in the pre CDK4/6 inhibitor era. In the FALCON study, PFS was superior with fulvestrant compared to AI in ET naïve MBC patients [77]. In the phase III EFFECT trial, efficacy of fulvestrant was similar to exemestane in patients who had progressed on a aromatase inhibitor [78]. However, in this study, fulvestrant was administered at 250 mg rather than the current standard of 500 mg. The superior efficacy of 500 mg dose over 250 mg was in the phase III CONFIRM study, which demonstrated a marginal improvement in both PFS and OS with the 500 mg dose [79]. Although fulvestrant has been frequently used as an ET partner with CDK4/6 inhibitors in phase III trials in both first- and second-line setting, the efficacy of single agent fulvestrant post progression on CDK4/6 inhibitor remains limited. Recent studies using fulvestrant as control arm in this setting have shown median PFS ranging from 1.9–4.7 months [20,21,80]. 

To improve upon the activity of fulvestrant, which is limited by poor bioavailability and weak permeation, and provide an easier route of administration, multiple oral SERDs have been tested in clinical trials with mixed results (Table 2). For instance, the pivotal randomized trials of amcenestrant and giredestrant failed to demonstrate a meaningful improvement in PFS, despite encouraging early-phase trial results, leading to discontinuation of their future development and further clinical trials by their respective pharmaceutical companies [81,82,83]. On the other hand, encouraging results for select patients were reported with elacestrant, which was tested in the phase 3 EMERALD study [19]. This was a phase III trial, that compared elacestrant to standard of care (SoC) ET (fulvestrant or an aromatase inhibitor) in 477 patients who had progressed on prior treatment with a CDK 4/6 inhibitor. Most patients in the SOC arm received fulvestrant (70%). More than 40% of patients in both arms had received two prior endocrine therapies and 20% had received chemotherapy for MBC. The study had coprimary end points of PFS in all patients and PFS in patients with an ESR1 mutations. Overall, there was a modest improvement in median PFS with elacesterant (2.8 vs. 1.9 months, hazard ratio 0.70, 0.55–0.88, *p* = 0.0018) with a significant proportion of patients in both arms progressing in the first six months suggesting endocrine resistance for nearly 50% patients in this setting. Six month and 12-month PFS rates were also improved with elacestrant (34.3% and 22% in elacestrant vs. 20.4% and 9.4% respective in SOC). Nausea was the most common side effect, with 35% experiencing nausea with elacestrant. Treatment discontinuations were observed in 3.4% of the patients with elacestrant compared to 0.9% in SOC arm.

Previous studies have suggested that about 30–40% patients develop an ESR1 mutation as a mechanism of resistance to ET under pressure from AI’s, a mechanism that can be potentially overcome by SERDs [31,86,87]. An ESR1 mutation was seen in 47.8% of patients in EMERALD. Retrospective analysis from SoFEA study and data from plasmaMATCH study suggest some activity of fulvestrant in patients with an ESR1 mutation [88,89]. Similarly, ESR1 mutation predicted for higher benefit in the EMERLALD study with a median PFS of 3.8 months with elacestrant and 6- and 12-month PFS rates of 41% and 27%. Of note, there was no benefit observed in ESR1 wild type and no OS benefit was observed overall. Data on the role of ESR1 mutation and length of prior CDK4/6 inhibitor treatment were recently presented, showing a 6 months PFS benefit (median 8.6 vs. 1.91 months, hazard ratio 0.41, 0.26–0.63) in patients with a ESR1 mutation who had remained on a prior CDK4/6 inhibitor for ≥12 months indicating that this might be the subgroup deriving most benefit from elacestrant [90]. On the basis of these data, the United States Food and Drug Administration (US FDA) approved elacestrant for postmenopausal patients with ER-positive, HER2-negative, ESR1-mutated ABC and disease progression after at least one line of ET [91]. Approval was also provided for Guardant360 CDx assay as a companion diagnostic to identify ESR1 mutation to select patients for this agent. 

Camizestrant is another oral SERD which has shown efficacy in this setting. The phase 2 SERENA-2 trial was meant to compare each of the 75 mg and 150 mg doses of camizestrant with fulvestrant with a primary end point of PFS [84]. The trial enrolled a less heavily pre-treated population compared to EMERALD with only 50% patients having received prior CDK4/6 inhibitors and no patient received more than one line of ET for MBC prior to enrolment. More importantly, prior exposure to fulvestrant or oral SERD was not allowed, a condition which was not mandatory in EMERALD study. Both doses of camizestrant produced a statistically significant improvement in PFS with median PFS for 75 mg being 7.2 months (3.7–10.9, hazard ratio 0.58, 0.41–0.81) and 150 mg being 7.7 months (5.5–12.9, hazard ratio 0.67, 0.48–0.92) compared to fulvestrant [median PFS 3.7 months [2,3,4,5,6]]. The response rates were modest (15% at 75 mg, 20% at 150 mg and 11% with fulvestrant). As with elacestrant, the benefits seem to be limited to patients with an ESR1 mutation. Currently, SERENA-6 study is comparing a switch to camizestrant 75 mg once daily vs. continuation on an AI in combination with CDK4/6 inhibitor in patients who have a detectable ESR1 mutation on ctDNA during first line treatment with an AI and CDK4/6 inhibitor [61] (Table 3). A similar strategy yielded promising results in the PADA-1 phase 3 trial when an AI was switched to fulvestrant on detection of an ESR1 mutation and may represent a biomarker based strategy to change treatment on development of resistance before clinical progression [92].

Preliminary data have also been presented for imlunestrant [96], and the ongoing phase 3 EMBER-3 study will further clarify its role in the treatment of patients with HR-positive MBC [62].

## 6. Proteolysis Targeting Chimeras (PROTACs)

PROTACs are a relatively new class of small molecules which possess the ability to target proteins for degradation via the ubiquitin-proteasome inhibition [97]. Unlike fulvestrant (which requires high affinity binding to ER for its action, thus requiring higher dose and more frequent administration and making it more prone to resistance due to point mutations in ER) the PROTAC ARV-471 (vepdegestrant) binds to E3 ubiquitin ligase and ER to trigger ER degradation via the proteasome pathway [98]. In breast xenograft models, ARV-471 yielded higher ER degradation and tumor growth inhibition than fulvestrant which led to its testing in clinical trials [99]. The results of the phase 2 dose expansion of ARV-471 (VERITAC) were recently presented [85] where a total of 71 patients with heavily pre-treated (median 3 lines) HR-positive MBC were treated at two dose levels of ARV-741 (200 mg and 500 mg QD). The overall clinical benefit rate (CBR), which was the primary end point, was 38% and was similar in both dose levels. Most of the responses were of stable disease with only two partial responses noted and with a median PFS of 3.7 months. A higher CBR (51.2%) was noted in patients with an ESR1 mutation, but numbers were small. A phase 3 study comparing ARV-471 to fulvestrant in patients who have previously progressed on a CDK4/6 inhibitor is currently ongoing and will help to define the role of this new class of compounds in the management of HR-positive MBC [63].

## 7. Targeting the PI3K/AKT/mTOR Pathway 

### 7.1. PI3K Inhibitors

Mutation in PIK3CA (which encodes for isoform 110 alpha of PI3K) lead to constitutive activation of PI3K and are seen in 30–50% of HR-positive MBC [100,101]. Most of these mutations are seen in exon 9 and exon 20, are considered early events in breast cancer pathogenesis [100,101,102] and can be detected either in plasma or tissue with good concordance [103,104]. Pan-PI3K inhibitors like buparlisib and taselesib were initially tested in MBC, but their development was halted due to significant toxicity concerns [105,106]. 

Alpelisib, an α-selective PI3K inhibitor, was the first PI3Kα inhibitor to demonstrate an improvement in PFS in patients with HR-positive HER2− MBC with activating PIK3CA mutations. The SOLAR-1 trial included postmenopausal women who were resistant to endocrine therapy, with disease progression on or after prior aromatase inhibitor. Only around 6% of patients previously received a CDK 4/6 inhibitor. Patients were randomized to receive alpelisib or placebo plus fulvestrant. The primary endpoint was PFS in the PIK3CA-mutated cohort. With a median follow-up of 20 months, the median PFS for the PIK3CA-mutated cohort was almost double with the addition of alpelisib, 11.0 vs. 5.7 months (hazard ratio 0.65 [95% CI 0.50–0.85]; [93]. The experimental arm was, however, burdened by a high rate of side effects, with key grade 3 and 4 toxicities being hyperglycemia in 36.6%, rash in 9.9% and diarrhea in 6.7% of the patients receiving alpelisib. In the final OS analysis, OS did not cross the pre-specified boundary (*p* ≤ 0.0161) for the PIK3CA-mutated cohort, although median OS was numerically prolonged by 7.9 months for patients in the alpelisib plus fulvestrant arm [94]. These interpretations are limited somewhat due to higher treatment discontinuations in the alpelesib and fulvestrant arm due to higher toxicity of the combination, leading to the risk of informative censoring. Subsequently reported patient reported outcomes showed a numerical worsening in quality of life with alpelesib compared to placebo, with deterioration in multiple symptom subscales possibly related to toxicity [107]. The data for alpelesib plus fulvestrant post CDK4/6 inhibitor progression are derived from a phase 2 open label BYLieve study [108]. Updated data from both cohort A (progression post CDK4/6 inhibitor and AI) and cohort B (progression post CDK4/6 inhibitor and fulvestrant) were presented at ASCO 2022, which showed a median PFS of 8.2 months (5.6–9.5) in cohort A and 5.6 months (3.7–7.1) in cohort B [109]. About 26% patients had >grade 3 side effects including hyperglycemia, rash and diarrhea [108]. Other α-selective mutant-degrading PI3K inhibitors (such as inavolisib) are currently being studied in this setting and data on efficacy and especially safety are awaited with great interest [64] (Table 1). Additionally, mutant-selective PI3K inhibitors like LOXO-783 and RLY-2608 are currently under clinical development and may allow to retain the activity of this class of drugs but reduce off-target toxicities [110,111].

### 7.2. AKT Inhibitors

Protein kinase B (AKT) is a key element of the PI3K/AKT/mTOR signaling pathway. AKT inhibitors in combination with ET showed preliminary clinically meaningful activity in early trials conducted in HR-positive HER2-negative MBC. However, the identification of biomarkers of response and resistance to AKT inhibition is crucial and still represents an unmet need [112,113].

The combination of capivasertib (an AKT inhibitor) and fulvestrant was explored in a randomized, placebo-controlled phase II trial (FAKTION) in postmenopausal women with HR-positive HER2-negative MBC progressing after or on an aromatase inhibitor. The primary endpoint was PFS. In the overall population, the addition of the AKT inhibitor to endocrine therapy provided a statistically significant 5.5-month gain in median PFS, 10.3 months in the capivasertib arm vs. 4.8 months in the control arm (hazard ratio 0.58 (95% CI 0.39–0.84); *p* = 0.004). Median OS in the experimental vs. placebo arms was 29.3 vs. 23.4 months (hazard ratio 0.66 (95% CI 0.45–0.97); *p* = 0.035) [114]. The benefits in this study were restricted to patients who had a mutation in the PIK3CA, AKT1 or PTEN; however, the analysis was exploratory due to limited number of patients in subgroups. The most common grade 3–4 adverse events were hypertension (32%), diarrhea (14%), and rash (20%) [115].

The results of the confirmatory phase III trial (CAPItello-291) of capivasertib and fulvestrant in HR-positive HER2- MBC patients after progression to an aromatase inhibitor-based therapy were recently presented [95] (Table 3). The study included pre/perimenopausal or postmenopausal ABC patients that progressed after aromatase inhibitor treatment, with or without a CDK4/6 inhibitor. The primary endpoint was PFS in the overall patient population and in the population of patients whose tumors had qualifying alterations in the AKT pathway (PIK3CA, AKT1 or PTEN genes). Of the 708 patients randomized, 41 percent had genetic alterations in the AKT pathway (31% being PIK3CA, 4.7% AKT and 5.2% PTEN) and 69 percent previously received a CDK4/6 inhibitor. The median PFS for the capivasertib/fulvestrant arm was 7.2 months compared with 3.6 months for placebo/fulvestrant (hazard ratio 0.60 (95% CI 0.51–0.61)). For patients in the AKT-altered population, the median PFS was 7.3 months for the capivasertib/fulvestrant group compared with 3.2 months for the placebo/fulvestrant group (hazard ratio 0.50 (95% CI 0.38–0.65)). In an exploratory analysis, the benefits were similar in patients with no mutation (including unknown status) in the AKT pathway (median PFS 7.2 months vs. 3.7 months, hazard ratio 0.70, 0.56–0.88). However, it should be noted that 16% of patients had an unknown mutation status and the hazard ratio for PFS excluding those patients was 0.79 (0.61–1.02). Further data from ctDNA based profiling is awaited to better understand the benefits in the unaltered group. The OS data remain immature but also showed a trend towards improvement in the overall population and AKT pathway mutated population. Major adverse events included diarrhea (72.4%, 9.3% grade 3–4) and rash (38%, grade 3–4 11.6%) with 9.3% patients discontinuing capivasertib due to adverse events [95]. Ongoing trials like FINER are testing other AKT inhibitors like ipatasertib post CDK4/6 inhibitor progression [65] and are expected to provide confirmatory evidence of efficacy of AKT inhibitors in this population (Table 1). Ongoing trials like CAPITELLO-292 are also looking at the efficacy of a combination of capivasertib, palbociclib and fulvestrant as first line treatment for HR + MBC [116].

### 7.3. mTOR Inhibitors

The mTOR signaling pathway regulates cell proliferation, autophagy, apoptosis, and is involved in malignant transformation. Better understanding of the complex regulatory mechanisms of the mTOR signaling pathway have been important in the development of mTOR inhibitors for treatment of cancer and in identifying predictors of response or resistance [117,118]. 

Everolimus has been tested with various endocrine partners. In the Pre0102 trial, addition of everolimus to fulvestrant improved median PFS (10.1 months to 5.3 months, hazard ratio 0.61 (0.40–0.92). Toxicities observed in the study included oral mucositis (53% vs. 12%), fatigue (42% vs. 22%), rash (38% vs. 5%), diarrhea (23% vs. 8%), hyperglycemia (19% vs. 5) and pneumonitis (17% vs. 0%) among others [119]. In the BOLERO-2 trial, the addition of everolimus to exemestane in postmenopausal women with HR-positive HER2-negative MBC who had progressed on aromatase inhibitor, improvement in median PFS with the combination was demonstrated (median PFS 10.6 months vs. 4.1 months according to central assessment, hazard ratio 0.36, 0.27–0.47) [76] (Table 3). However, there was a higher rate of discontinuation of everolimus due to adverse events, 29% compared to 5% in the control arm, leading to informative censoring that likely influenced these results [120]. Furthermore, an update of the trial reported no improvement in OS with a median of 31.0 months in the combination group compared to 26.6 months in the group receiving Exemestane and placebo (*p* = 0.14) [121]. Everolimus was also evaluated in combination with exemestane compared to exemestane or capecitabine alone in the BOLERO-6 trial. Consistent with the BOLERO-2 study, it showed evidence of superiority in median PFS for everolimus plus exemestane vs. everolimus alone but not vs. capecitabine alone [122]. Moreover, serious adverse events were more common with everolimus exemestane (36%) compared to capecitabine (29%), again raising questions about toxicity of this potential chemotherapy free approach. An ongoing phase 3 study (eVERA) is currently evaluating the combination of oral SERD giredestrant and everolimus compared to everolimus and exemestane in patients who have previously progressed on a CDK4/6 inhibitor (Table 1). However, given the previous negative data for single agent giredestrant and safety concerns with everolimus, it is critical to be able to identify which patients would benefit from these drugs [66].

## 8. PARP Inhibitors

Two randomized phase III studies were conducted to evaluate the efficacy of PARP inhibitors (olaparib and talazoparib) in germline BRCA mutated MBC patients in the pre CDK 4/6 inhibitor era [123,124,125,126]. About half of the patients in both these trials had HR-positive disease, and all patients had received at least one prior chemotherapy regimen. The trials effectively excluded patients resistant to platinum, and platinum was not allowed as one of the investigator choice chemotherapy regimens. Overall, both trials demonstrated an improvement in ORR and PFS (median improvement 3 months) with the PARP inhibitor but failed to demonstrate an improvement in OS [124,125]. How prior CDK4/6 inhibitor treatment would modify these treatment effects remains unknown. However, for selected patients with germline BRCA mutation, PARP inhibitors remain a reasonable alternative to chemotherapy beyond CDK4/6 inhibitors in the endocrine refractory setting [127].

## 9. Role of Genomic Testing to Select Treatment after CDK4/6-Inhibitors

The approval of elacestrant post progression on CDK4/6 inhibitors for patients with a ESR1 mutation has added another layer of complexity to genomic testing and its role for patients with HR-positive MBC. Previously, alpelisib was approved for patients with a PIK3CA mutation, which could be detected on either tissue or blood with good concordance and at any time point in the disease course since it is a founder mutation [100,101,102,103,104]. However, ESR1 mutation usually develops as a resistance mechanism to an AI and prevalence of ESR1 mutation at the time of primary diagnosis is low (usually <5%, <1% in AI naïve MBC) [44,124,128]. Moreover, the prevalence of ESR1 mutations increases over time in patients with MBC and a recent liquid biopsy assay to detect this alteration is required rather than relying on a remote test [129,130]. 

Therefore, for the optimal selection of treatment after CDK4/6-inhibitors, next-generation sequencing with tissue or preferentially liquid biopsy is recommended, to understand the most updated ESR1 (and PIK3CA) status of the disease, which is key for treatment decisions. 

It is worth noticing that testing by either method does add cost and complexity and the clinical implications of using one method over the other remain to be defined. Moreover, deeper knowledge regarding the complexities of ESR1 mutations is warranted. The recent plasmaMATCH study, for instance, emphasized the importance of ESR1 variant allele frequency (VAF) when no responses to fulvestrant were seen when VAF was <50% [89]. The correlation of VAF and outcomes with oral SERDs thus needs further study.

## 10. How to Approach a Patient with Hormone Receptor Positive MBC Who Has Progressed on First Line CDK4/6 Inhibitor and ET in 2023?

**Figure 1:**
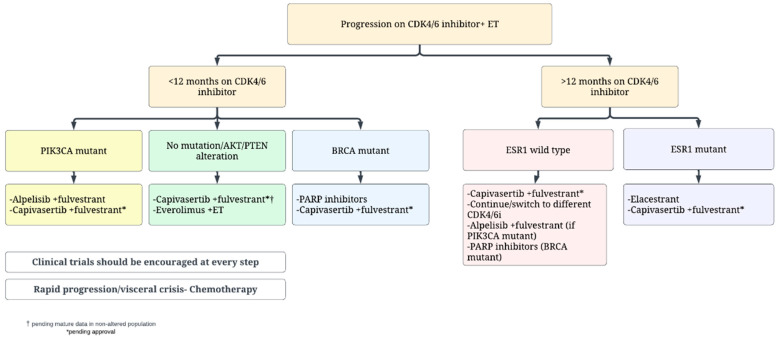
Proposed second line treatment algorithm for patients with hormone receptor positive MBC.

Several aspects need to be considered to select the most adequate treatment after progression on first line CDK4/6 inhibitor plus ET (Figure 1). Among these, key factors are represented by prior duration of exposure to CDK4/6 inhibitors, patient preference, comorbidities, recent somatic mutation status including ESR1, PIK3CA and germline BRCA1/2 results. Although elacestrant has been approved in all patients with an ESR1 mutation, the majority of the benefit seems to be derived in patients who are endocrine sensitive with a prolonged exposure to prior CDK 4/6 inhibitors. Therefore, the adoption of second line elacestrant seems best suited for patients with ESR1-mutant disease that experienced prolonged benefit (at least 12 months) from prior CDK4/6-inhibitors.

In the presence of a PIK3CA mutation, the use of fulvestrant plus alpelisib can be discussed with patients. The previously mentioned issues with toxicities of the regimen may turn this choice less preferrable in the presence of active alternatives. Among these, capivasertib may soon achieve regulatory approval based on the data of CAPITELLO-291 study [95]. If approved, it might be reasonable to use second line capivasertib plus fulvestrant, particularly for patients with PIK3CA, AKT or PTEN alterations, who seemed to derive a major benefit in the trial; the role of this combination in patients without mutations will require elucidation in future updates of the CAPITELLO-291 study. Fulvestrant plus everolimus also represents an available option in this setting, although with little data after progression to CDK4/6 inhibitors, and with non-negligible toxicities.

For patients with a germline BRCA mutation, it is reasonable to discuss a PARP inhibitor (either olaparib or talazoparib) as second or third line as a non-chemotherapy option after discussion of risks, benefits and limitations of no OS benefit yet demonstrated [124,125].

The continuation of CDK4/6 inhibitor beyond progression remains controversial given the conflicting data discussed above [21,22,23,24,25,26,27,28,29,30,31,32,33,34,35,36,37,38,39,40,41,42,43,44,45,46,47,48,49,50,51,52,53,54,55,56,57,58,59,60,68,69,70,71,72,73,74,75,76,77,78,79,80]. This approach is not standard clinical practice and the mentioned research studies are likely to provide more information about the efficacy of this approach and on patient selection. The signal for addition of immunotherapy in such patients from PACE trial is intriguing and clinical trials considering this approach and addition of other targeted agents to CDK4/6 inhibitors plus ET should be considered in these patients (Table 1).

Lastly, for patients with rapid clinical progression with impending visceral crisis, single agent chemotherapy remains the preferred approach with the choice of agent dictated by patient preference and side effect profile. These patients are also likely to benefit from antibody drug conjugates like trastuzumab deruxtecan or sacituzumab govitecan, and enrollment on clinical trials testing these strategies should be encouraged (Table 1).

## 11. Conclusions

The treatment of patients who experience disease progression on CDK4/6 inhibitors and ET remains challenging. Multiple novel biological and endocrine therapies have shown promise in this setting. A subset of patients, defined by the presence of an ESR1 mutation and a prolonged benefit from prior CDK4/6 inhibition, derive benefit from elacestrant, which is now an approved treatment option in the US. Adding the AKT inhibitor capivasertib to fulvestrant achieved an encouraging benefit in PFS in patients with progression while on CDK4/6 inhibitors and may soon become an available treatment option. Continuation of CDK4/6 inhibitors beyond progression remains controversial and more data is required before this approach can be considered standard. Despite the recent advances in treatment, outcomes for these patients remain suboptimal and enrolment into ongoing clinical trials is strongly encouraged at every step. Progression on CDK4/6 inhibitor therapy is a major change, demarcating nearly half of the survival time in a patient’s life facing HR-positive, HER2-negative MBC. It is an excellent time for the clinician to take a step back, reassess tumor biology and initiate discussions about the patients’ goals and preferences. Patient shared decision making is always encouraged.

## Figures and Tables

**Table 1:** Ongoing/completed 2nd line and beyond clinical trials.

Trial No	Phase	Regimen	Status	Purpose
NCT03519178 [56]	½	PF-06873600 alone and in combination with endocrine treatment	Active, not recruiting	Evaluating dual CDK2 and CDK4/6 inhibitor for multiple tumor types including HR + ABC after prior CDK4/6 inhibitor
NCT04318223 [52]	2	Palbociclib + Fulvestrant	Recruiting	Evaluating the efficacy and safety of palbociclib plus fulvestrant after failure of a combined treatment of hormonal therapy (aromatase inhibitor or tamoxifen ± LHRHa) plus a CDK4/6 inhibitor, in women with HR+ and HER2- LABC or MBC
NCT03809988 (PALMIRA) [54]	2	Palbociclib rechallenge + ET	Completed	Evaluate the efficacy and safety of continuation of palbociclib + 2nd line endocrine therapy in HR + /HER2- ABC patients who had clinical benefit during 1st line palbociclib.
NCT03280563 (MORPHEUS HR+ BC) [57]	1B/II	Atezolizumab + Bevacizumab/Entinostat, Exemestane/Fulvestrant Ipatasertib/Tamoxifen Abemaciclib	Active, not recruiting	Randomized umbrella study evaluating the efficacy and safety of multiple immunotherapy-based treatment combinations in patients with hormone receptor-positive HER2-negative breast cancer
NCT03099174 [58]	I	Xentuzumab + abemaciclib	Active, not recruiting	Study testing anti IGF ½ antibody Xentuzumab in combination with abemaciclib and fulvestrant in both treatment naïve (cohort D) and pre-treated with CDK 4/6 inhibitor breast cancer patients (Cohort F)
NCT02684032 [59]	IB	Gedatolisib + Palbociclib + fulvestrant/letrozole	Completed	Evaluated the safety and MTD of the dual mTOR/PI3K inhibitor gedatolisib in multiple combinations in treatment-naive patients (cohort A), CDK4/6i-naive patients (cohort B), and CDK4/6i-pretreated patients (cohorts C/D)
NCT03238196 [60]	IB	Erdafitinib + Palbociclib + Fulvestrant	Active, not recruiting	Evaluates the safety and tolerability of the FGFR inhibitor erdafitinib in combination with palbociclib/fulvestrant in CDK4/6i-pretreated patients with FGFR-amplified MLBC
NCT04964934 [61]	III	Camizestrant plus Palbociclib vs. AI + Palbociclib	Active, recruiting	Evaluates whether switching to Camizestrant on detection of ESR1 mutation in ctDNA improves PFS compared to continuation on CDK4/6 inhibitor plus AI in first line setting
NCT04975308 [62]	III	Imlunesterant vs. imlunesterant + abemaciclib vs. investigator choice of endocrine therapy	Active, recruiting	Three arm study looking at efficacy of Imlunestrant and combination of Imlunestrant plus abemaciclib compared to ET of investigator choice in patients who have progressed on previous endocrine therapy and a CDK4/6 inhibitor
NCT05654623 [63] (VERITAC-2)	III	ARV-471 vs. fulvestrant	Not yet recruiting	Phase three study to evaluate the efficacy of ARV-471 in patients progressed on prior endocrine therapy for advanced breast HR+ breast cancer. Prior chemotherapy not allowed
NCT03006172 [64]	I	Arm C: Inavolisib + Letrozole Arm D: Inavolisib + Fulvestrant	Recruiting	Arm C and arm D testing dose escalation, safety and efficacy of Inavolisib in patients with CDK4/6 inhibitor pretreated HR + MBC
NCT04650581 [65]	III	Ipatasertib + fulvestrant vs. fulvestrant alone	Recruiting	Evaluate the efficacy of ipatasertib in patients with HR + MBC after progression on prior CDK4/6 inhibitor and AI
NCT05306340 [66]	III	Giredestrant plus everolimus vs. everolimus plus exemestane	Recruiting	Evaluate the efficacy of Girdestrant (oral SERD) + everolimus compared to everolimus exemestane in patients who have previously progressed on a CDK4/6 inhibitor
NCT04494425 [67] (DB-06)	III	T-DXD vs. investigator choice chemotherapy	Recruiting	Phase three study to evaluate the efficacy of T-DXD vs. Investigator choice chemotherapy in chemotherapy naïve patients with HR + Her-2 low or ultra-low breast cancer

**Table 2:** Positive studies of oral SERDs (including PROTACs).

SERD	Trial	Phase	Experimental Arm	Control Arm	Prior CDK4/6i	Prior Fulvestrant	ESR1 Mutations	Grade 3 Toxicity	mPFS, Months (95% CI)	ESR1 Mutant mPFS, Months (95% CI)
Elacestrant [19]	EMERALD Trial	3	elacestrant 400 mg	endocrine monotherapy	100%	29.30%	47.80%	2.5% vs. 0.9%	2.8 (NR)	3.8 (2.2–7.3)
Camizestrant [84]	SERENA-2	2	camizestrant (75,150 and 300 mg)	fulvestrant	51%	0%	38%	1.4% and 2.7%	7.2 (3.7–10.9), 7.7 (5.5–12.9)	6.3 (3.4–12.9), 9.2 (3.7–12.9)
ARV-471 [85]	VERITAC	2	ARV-471 200 mg orally QD		100%	79%	57.70%	21%	3.7 (1.9–8.3)	5.7 (3.6–9.4)

**Table 3:** Positive studies of PI3K/AKT/mTOR inhibitors.

PI3K/AKT/mTOR Inhibitor	Trial	Phase	Experimental Arm	Control Arm	Prior CDK4/6i	Grade 3 Toxicity	mPFS, Months	mOS, Months
Alpelisib + fulvestrant [93,94]	SOLAR-1	3	Alpelesib (300 mg) + fulvestrant	Fulvestrant	6%	76% vs. 35%	11.0 vs. 5.7 months	39.3 vs. 31.4 months (NS)
Capivasertib + fulvestrant [95]	CAPITELLO-291	3	Capivasertib (400mg 4 days on, 3 d off)	Fulvestrant	70%	16% vs. 8%	7.2 vs. 3.6 months	immature
Everolimus + exemestane [58,59,60,61,62,63,64,65,76,77,78,79,80,81,82,83,84,85,86,87,88,89,90,91,92,93,94,95,96,97,98,99,100,101,102,103,104,105,106,107,108,109,110,111,112,113,114,115,116,117,118,119,120,121]	BOLERO-2	3	Everolimus 10 mg	Exemestane	0	11% vs. 1%	10.1 vs. 4.3 months	31 vs. 26.6 months (NS)
